# Draft genome sequence of active gold mine isolate *Pseudomonas iranensis* strain ABS_30

**DOI:** 10.1128/MRA.00849-23

**Published:** 2023-11-15

**Authors:** Adetomiwa A. Adeniji, Ayansina S. Ayangbenro, Olubukola O. Babalola

**Affiliations:** 1 Food Security and Safety Focus Area, Faculty of Natural and Agricultural Sciences, North-West University, Mmabatho, South Africa; 2 Center for Epidemic Response and Innovation, School of Data Science and Computational Thinking, Stellenbosch University, Cape Town, South Africa; University of Maryland School of Medicine, Baltimore, Maryland, USA

**Keywords:** bioremediation, biosynthetic clusters, genome sequence, gold mine, *Pseudomonas iranensis*, secondary metabolites

## Abstract

*Pseudomonas iranensis* ABS_30, isolated from gold mining soil, exhibits metal-resistant properties valuable for heavy metal removal. We report the draft genome sequencing of the *P. iranensis* ABS_30 strain, which is 5.9 Mb in size.

## ANNOUNCEMENT

*Pseudomonas* species thrive ubiquitously in soil, water, humans, and plants (1). *Pseudomonas* have extensive metabolic adaptability and genetic diversity, allowing them to make and utilize a diverse range of chemical compounds (2, 3). While some Pseudomonads are disease-causing (4, 5), others have beneficial properties (6, 7). Following rpoD-based identifications, the genus recently underwent phylogenetic reclassification. *Pseudomonas iranensis* sp. was reassigned into the *Pseudomonas koreensis* subgroup (8, 9). Little is known about this species, including its pathogenic or beneficial properties (10).

*P. iranensis* ABS_30 was isolated in June 2016 from active gold mine soil samples at depths ranging from 10 to 30 cm in Vryburg (S 26.1675545 E 25.255411), North West Province, South Africa as described by Ayangbenro (11). Briefly, 1 g of soil sample collected was serially diluted and 10^−4^ plated out on Luria-Bertani (LB) agar previously supplemented with 50 mg/L of CdSO_4_ used for resistant test. The plates were incubated at 30°C for 48 h. The resistant isolate was thereafter evaluated for its ability to grow at different metal concentrations (100–1,000 mg/L) on LB agar plates (11). Using a ZR soil microbe DNA mini prep DNA extraction kit from Zymo Research (CA, USA), we extracted genomic DNA of the isolate from pure cultures grown on LB agar. Genome sequencing of isolate *P. iranensis* ABS_30 was done at Novogene Co. Ltd., Singapore. A paired-end (PE) sequencing library was prepared from the DNA sample using the Illumina Nextera DNA Flex library preparation kit. The PE Illumina library was loaded onto the NovaSeq 6000 (2 × 150 bp) instrument for cluster generation and sequencing.

The reads totaling 15,212,600 were retrieved in FASTQ format. FastQC (v1.2.2) on the Kbase server was used to assess read quality (12, 13). Trimmomatic (v0.36) and Cutadapt (v1.18) apps on the KBase server were used to trim reads and remove adaptor sequences, respectively (12). GTDBtk (v.1.7.0) was used for taxonomy classification (13). Isolate ABS_30 was classified as *P. iranensis* with closest match type-strain *P. iranensis* SWRI54 (GCF_014268585.2/). The assemblage of reads into contigs with SPAdes (v3.15.3) was done on the KBase platform (12). The contigs from KBase platform were uploaded on online servers of NCBI Prokaryotic Genome Annotation Pipeline (PGAAP v6.5) for automated annotation and comparison. Biosynthetic gene clusters were identified using AntiSMASH (v6.1.1) (14). Default settings were used for all bioinformatics analyses.

The 5.9 Mb *P*. *iranensis* ABS_30 genome sequence has 34 scaffolds and 44 contigs (Table 1). As predicted by AntiSmash, the organism’s genome contains redux-factor proclusters, post-translationally modified peptide products (RiPP), and other crucial gene clusters involved in the production of beneficial secondary metabolites (e.g., lankacidin C). The use of this genomic data could lead to a better understanding of *P. iranensis* sp.’s genetic diversity, metabolic features, and biotechnological prospects.

**TABLE 1:** Genome annotation features of *P. iranensis* ABS_30

Parameter	Value
Genome size	5.9 Mb
Number of contigs	34
Number of scaffolds	44
tRNA	58
rRNA	4
Plasmids	0
GC content	60.10%
Hypothetical proteins	1,167
Contig L50	7
Contig N50	409.3 kb
CDS	5,393

## Data Availability

The draft whole genome shotgun project has been deposited at DDBJ/ENA/GenBank under the accession number JAVCAN000000000.1 (https://www.ncbi.nlm.nih.gov/nuccore/JAVCAN000000000.1) and the assembly accession number GCA_030769685.1 (https://www.ncbi.nlm.nih.gov/datasets/genome/GCA_030769685.1/). The version described in this paper is version JAVCAN000000000. The project data are available under BioProject accession number PRJNA1004443 (https://www.ncbi.nlm.nih.gov/bioproject/PRJNA1004443) and BioSample accession number SAMN36942924 (https://www.ncbi.nlm.nih.gov/biosample/SAMN36942924).
